# Monogenic Lupus: A Developing Paradigm of Disease

**DOI:** 10.3389/fimmu.2018.02496

**Published:** 2018-10-30

**Authors:** Jessie M. Alperin, Lourdes Ortiz-Fernández, Amr H. Sawalha

**Affiliations:** ^1^Division of Rheumatology, Department of Internal Medicine, University of Michigan, Ann Arbor, MI, United States; ^2^Center for Computational Medicine and Bioinformatics, University of Michigan, Ann Arbor, MI, United States

**Keywords:** lupus, monogenic, familial, genetic, mendelian

## Abstract

Monogenic lupus is a form of systemic lupus erythematosus (SLE) that occurs in patients with a single gene defect. This rare variant of lupus generally presents with early onset severe disease, especially affecting the kidneys and central nervous system. To date, a significant number of genes have been implicated in monogenic lupus, providing valuable insights into a very complex disease process. Throughout this review, we will summarize the genes reported to be associated with monogenic lupus or lupus-like diseases, and the pathogenic mechanisms affected by the mutations involved upon inducing autoimmunity.

## Introduction

Systemic lupus erythematosus (SLE or lupus) is a complex multisystem disease whose underlying disease mechanism continues to be a topic of intense research. SLE can affect many organs including the kidneys, skin, joints, lungs, cardiovascular system, central nervous system, and hematopoietic system. As with most complex diseases, the etiology of SLE is incompletely understood, however, cumulative evidence has pointed to the involvement of both genetic and epigenetic mechanisms (1, 2). Multiple genetic variants associated with lupus susceptibility have been identified through genome-wide association studies (GWAS). Support for a genetic component of lupus can be realized from twin studies. Concordance rate of lupus in monozygotic and dizygotic twins has been reported to be 24 and 2%, respectively, demonstrating a role for genetic susceptibility in lupus (3). In parallel, monozygotic twin studies have also provided evidence highlighting the relevance of DNA methylation changes (4). At the same time, non-genetic factors such as viral infections or exposure to ultraviolet (UV) light among others are clearly involved, as suggested by incomplete concordance in monozygotic twins.

Patients with childhood onset SLE usually present with a more severe phenotype and have an increased frequency of glomerulonephritis, cytopenias, neuropsychiatric disease, cutaneous manifestations, anti-dsDNA antibodies, and hemolytic anemia (5). It can be presumed that in early onset disease, genetic factors may play a more important role than environmental and hormonal factors (5). Monogenic lupus is a form of SLE that typically presents early in life, usually at < 5 years of age, with severe disease manifestations. This form of lupus is caused directly by a genetic variant in a specific gene. Monogenic lupus represents a collection of distinct genetic abnormalities causing similar clinical features and resulting in autoantibody production. In particular, consanguinity presents a significant increased risk for monogenic lupus and should prompt consideration in patients with familial SLE. Though monogenic lupus accounts for only a small subset of lupus patients, it provides significant insight into the cause and mechanisms of lupus, and potential treatment strategies.

**Figure 1:**
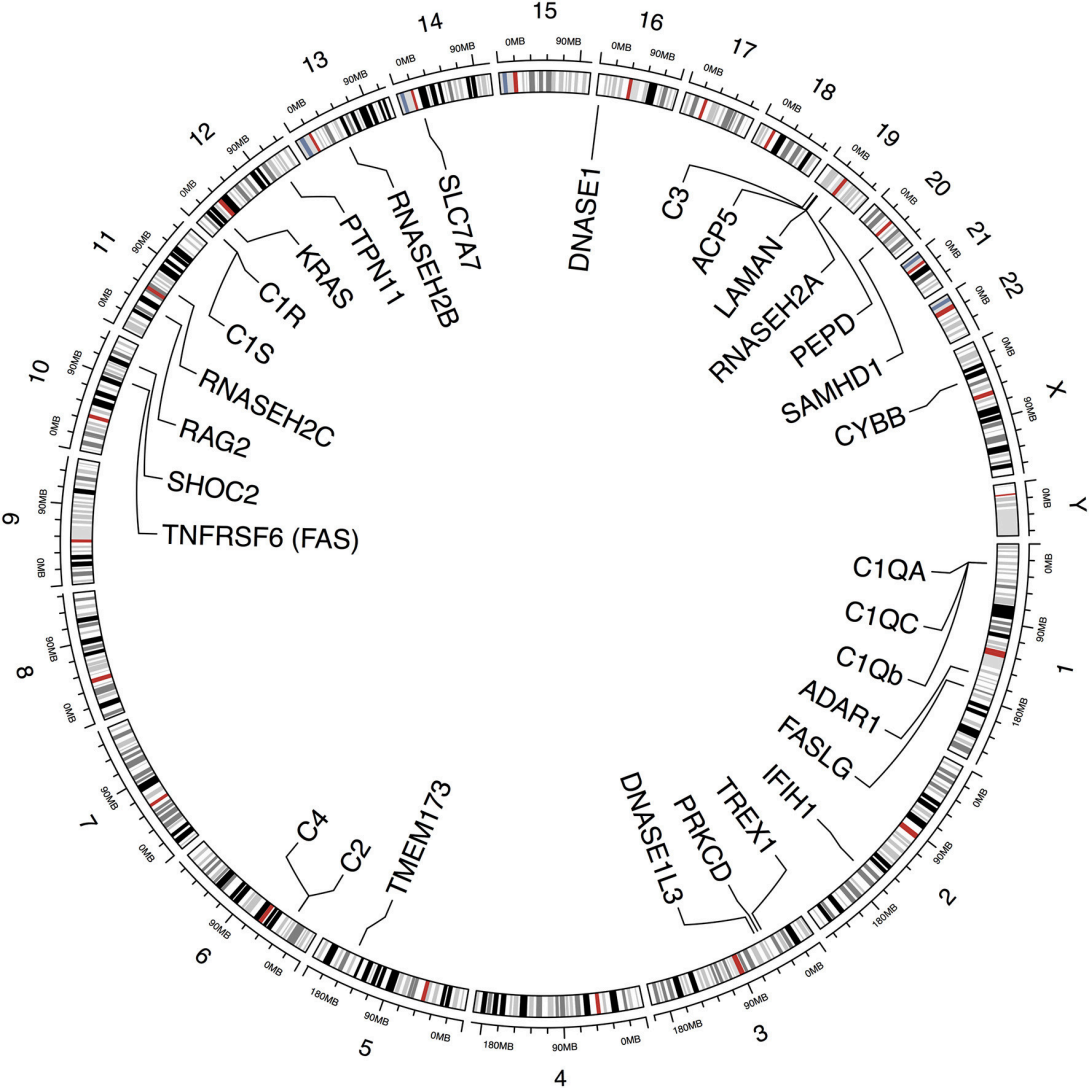
Schematic representation depicting genes and chromosomal locations previously identified to be implicated in monogenic lupus. This figure was produced using ‘circlize' in R (http://cran.r-project.org/web/packages/circlize/).

In the last years, large achievements understanding the genetic component of SLE have been accomplished. More than 80 loci have been reported to be associated with susceptibility in polygenic lupus, and a considerable number of monogenic causes of SLE and lupus-like syndromes are emerging due to the evolution of new sequencing techniques that can identify rare genetic variations across the entire genome. Through this review, we will explore the implications on the disease pathogenesis of the genes that have been found to cause monogenic lupus or lupus-like phenotype (Figure 1, Table 1).

**Table 1:** List of genes identified to cause monogenic lupus or lupus-like diseases.

***Locus***	**Gene name**	**Gene Location**	**Protein**	**Inheritance**	**Pathway**	**Phenotype**	**References**
*ACP5*	Acid phosphatase 5, tartrate resistant	19p13.2	TRAP	AR	Nucleic acid sensing and degradation Type I IFN	SPENCD SLE	(6)
*ADAR1*	Adenosine deaminase, RNA specific	1q21.3	Adenosine deaminase, RNA specific	AR/AD	Type I IFN	AGS SLE	(7, 8)
*C1QA*	Complement C1q A chain	1p36.12	C1q	AR	Complement	Complement deficiencies SLE	(9)
*C1QB*	Complement C1q B chain	1p36.12	C1q	AR	Complement	Complement deficiencies SLE	(9)
*C1QC*	Complement C1q C chain	1p36.12	C1q	AR	Complement	Complement deficiencies SLE	(9)
*C1R*	Complement C1r	12p13.31	C1r	AR	Complement	Complement deficiencies SLE	(10)
*C1S*	Complement C1s	12p13.31	C1s	AR	Complement	Complement deficiencies SLE	(11)
*C2*	Complement C2	6p21.33	C2	AR	Complement	Complement deficiencies SLE	(12)
*C4A*	complement C4A	6p21.33	C4	AR	Complement	Complement deficiencies SLE	(13)
*C4B*	complement C4B	6p21.33	C4	AR	Complement	Complement deficiencies SLE	(13)
*CYBB*	Cytochrome b-245 beta chain	Xp21.1-p11.4	NADPH oxidase 2	X-linked	Phagocytosis	Chronic granulomatous disease	(14)
*DNASE1*	Deoxyribonuclease 1	16p13.3	DNASE1	AD	Nucleic acid sensing and degradation	SLE	(15)
*DNASE1L3*	Deoxyribonuclease 1 like 3	3p14.3	DNASE1L3	AR	Nucleic acid sensing and degradation	SLE	(16)
*FAS or TNFRSF6*	Fas cell surface death receptor	10q23.31	FAS	AD	Apoptosis	ALPS	(17)
*FASL*	Fas ligand	1q24.3	FASL	AD	Apoptosis	ALPS	(18)
*IFIH1*	Interferon induced with helicase C domain 1	2q24.2	IFIH1	AD	Type I IFN	AGS SLE	(19)
*ISG15*	ISG15 ubiquitin-like modifier	1p36.33	ISG15	AR	Type I IFN	AGS	(20)
*KRAS*	KRAS proto-oncogene, GTPase	12p12.1	KRAS	AD	RAS-MAPK signaling	Noonan syndrome	(21)
*LAMAN or MAN2B1*	Mannosidase alpha class 2B member 1	19p13.13	Lysosomal Alpha-mannosidase	AR	Metabolism of carbohydrates	Alpha-mannosidosis	(22)
*PEPD*	Peptidase D	19q13.11	PEPD	AR	Aminopeptidase activity	Prolidase defiency	(23)
*PRKCD*	Protein kinase C delta	3p21.1	PRKCD	AR	Self-tolerance	SLE	(24)
*PSMA3*	Proteasome subunit alpha 3	14q23.1	PSMA3	AD	Proteasome	CANDLE	(25)
*PSMB4*	Proteasome subunit beta 4	1q21.3	PSMB4	AD	Proteasome	CANDLE	(25)
*PSMB8*	Proteasome subunit beta 8	6p21.32	PSMB8	AD	Proteasome	CANDLE	(25)
*PTPN11*	Protein tyrosine phosphatase, non-receptor type 11	12q24.13	PTPN11	AD	RAS-MAPK signaling	Noonan syndrome	(26)
*RAG2*	Recombination activating 2	11p12	RAG2	AR/AD	Self-tolerance	SLE	(27)
*RNASEH2A*	Ribonuclease H2 subunit A	19p13.13	RNASEH2 Complex	AR	Nucleic acid sensing and degradation	AGS	(28)
*RNASEH2B*	Ribonuclease H2 subunit B	13q14.3	RNASEH2 Complex	AR	Nucleic acid sensing and degradation	AGS	(28)
*RNASEH2C*	Ribonuclease H2 subunit C	11q13.1	RNASEH2 Complex	AR	Nucleic acid sensing and degradation	AGS	(28)
*SAMHD1*	SAM and HD domain containing deoxynucleoside triphosphate triphosphohydrolase 1	20q11.23	SAMHD1	AR	Type I IFN	AGS SLE FCL	(29, 30)
*SHOC2*	SHOC2, leucine rich repeat scaffold protein	10q25.2	SHOC2	AD	RAS-MAPK signaling	Noonan syndrome	(31)
*SLC7A7*	Solute carrier family 7 member 7	14q11.2	SLC7A7	AR	Amino acid transporter	Lysinuric protein intolerance	(32)
*TMEM173*	transmembrane protein 173	5q31.2	STING	AD	Type I IFN	SAVI	(33)
*TREX1*	three prime repair exonuclease 1	3p21.31	TREX1	AD	Nucleic acid sensing and degradation	AGS FCL	(34)

*AGS, Aicardi-Goutières Syndrome; AD, autosomal dominant; ALPS, autoimmune lymphoproliferative syndrome; AR, autosomal recessive; CANDLE, chronic atypical neutrophilic dermatosis with lipodystrophy and elevated temperature; FCL, familial chilblain lupus; SAVI, STING-associated vasculopathy with onset in infancy; SLE, systemic lupus erythematosus; SPENCD, Spondyloenchondrodysplasia*.

## Interferonopathies

Although the clinical manifestations and severity observed are different among patients, the interferonopathies are a wide group of complex genetic disorders with a common pathogenic mechanism characterized by imbalance of interferon (IFN) mediated immune responses. Given that studies have repeatedly identified an increased blood IFN signature in SLE patients (35, 36), it is not surprising that clinical features of some of the diseases classified as interferonopathies overlap with systemic lupus erythematosus. Consequently, some of these diseases, such as Aicardi-Goutières Syndrome (AGS) and Spondyloenchondrodysplasia (SPENCD) can also be considered as forms of monogenic lupus or lupus-like syndromes.

### Aicardi-goutières syndrome

Aicardi-Goutières Syndrome is a genetic syndrome caused by multiple genetic defects. The disease phenotype resembles a congenital viral infection and many patients develop lupus as a feature of the syndrome. AGS develops in young patients, typically before 6 months of age and is characterized by encephalopathy that is usually associated with calcification of the basal ganglia and white matter changes on brain imaging (37). Frequently, there is cutaneous involvement and, in particular, chilblains. Patients with AGS have been shown to have multiple IgG autoantibodies particularly directed against nuclear antigens, basement membrane components, gliadin, and brain endothelial cells and astrocytes. Patient with AGS develop elevated type I IFN levels in both serum and cerebral spinal fluid (7). These observations suggest that genetic defects in RNA or DNA clearance result in increased type I IFN production and interferon stimulated genes (ISGs), and then lead to autoimmunity. AGS is associated with mutations in multiples genes and many of these overlap with various forms of monogenic lupus, suggesting a spectrum of disease likely influenced by the location of the mutation as well as environmental factors (7).

Mutations of *TREX1* gene have been identified in AGS. Approximately 25% of AGS patients have a *TREX1* mutation. Patients with AGS due to a *TREX1* mutation have a prototypical disease phenotype. A review of autoimmune features in AGS patients showed that approximately 60% of patients with a *TREX1* variant had at least one autoimmune feature: thrombocytopenia, leukopenia, antinuclear antibodies (ANA), skin lesions, oral ulceration, arthritis, anti-dsDNA antibodies, or antibodies to extractable nuclear antigens (ENA) (34). Persisting severe physical and intellectual disability is frequent. In the large majority, patients will have no purposeful gross motor, hand or communication function. About a third of patients with a *TREX1*-related AGS present in the neonatal period with thrombocytopenia, hepatosplenomegaly, and a transaminitis in addition to neurological disease (7). For reasons that are not understood, these extra-neurological features often resolve within the first year of life. A broad spectrum of mutations across *TREX1* have been associated with different immune-mediated diseases. Most patients with AGS show biallelic mutations within *TREX1* with autosomal recessive inheritance, which usually causes a complete loss of protein function. However, some heterozygous mutations have also been identified in individuals diagnosed with AGS (34). Early-onset familial chilblain lupus (FCL) is a rare form of cutaneous lupus which results in cold-induced severe discoloration of hands, feet, and ears, where the lesions frequently ulcerate. Most of these patients have heterozygous mutations with autosomal dominant inheritance (34). In addition, heterozygous mutations in *TREX1* with autosomal dominant inheritance has been linked to retinal vasculopathy with cerebral leukodystrophy (RVCL) (38). Interestingly, a deleterious homozygous variant of this gene has been recently identified in a patient with cerebral SLE (39), and, it is worth noting that several single nucleotides polymorphisms (SNPs) in *TREX1* have been found associated with common forms of SLE in different populations (40–42). This gene, located on chromosome 3, encodes a protein with exonuclease activity which is an IFN-inducible protein responsible for degradation of genomic DNA in response to DNA damage. Therefore, it plays an important role in the immune response to single-stranded (ss)-DNA and dsDNA (43), and maintains immune tolerance to cytosolic self-DNA (44). When TREX1 is dysfunctional, the cytosolic DNA does not get degraded which constitutes a damage-associated molecular pattern (DAMP). This activates the cGAS-STING-mediated type I IFN response and systemic inflammation (44). TREX1 deficiency is thought to trigger autoimmunity through the accumulation of self DNA in the cytosol. These are sensed by cyclic GMP-AMP (cGAMP) synthase (cGAS). cGAMP is a ligand for stimulator of IFN genes protein (STING), which leads to the production of type I IFN (43). On the other hand, TREX1 is a DNase component of the SET complex which is involved, among other mechanisms, in apoptosis (45). Altogether, these data provide different pathomechanisms for the involvement of TREX1 dysfunction in SLE.

Patients with *IFIH1* (interferon induced with helicase C domain 1) gain of function mutations can develop early onset SLE and AGS-like disease, including musculoskeletal involvement and Jaccoud's arthropathy (46). *IFIH1* gene, which encodes MDA5 (melanoma differentiation-associated protein 5), is a cytoplasmic RNA receptor that binds cytoplasmic double-stranded RNA. IFIH1 belongs to the RIG-I–like family which is part of the pathway responsible for activating type I interferon signaling (19). The gain of function mutation in *IFIH1* gene leads to activated dendritic cells and macrophages, which are the primary producers of IFN-alpha in response to nucleic acid (47). This leads to the activation of T cells and production of autoantibodies (48). It is interesting to note that polymorphisms in *IFIH1* have been reported in patients with inflammatory myopathies and anti-MDA5 antibodies are seen in some patients with amyopathic dermatomyositis (49). Remarkably, a recent study revealed that these patients showed a high activity of type I IFN system. Although the mechanisms are still unclear, the study detected high levels of transcripts of IFN-associated sensors and several IFN-inducible genes were up-regulated in these patients (50).

SAMHD1 (SAM domain and HD domain-containing protein 1) is a dGTP-dependent triphosphohydrolase responsible for the regulation of intracellular levels of deoxynucleoside triphosphates (dNTPs), the building blocks of DNA synthesis (51, 52). In unaffected individuals, SAMHD1 promotes cell stability and prevents reverse transcription of retroviruses. Deficiency of SAMHD1 results in unbalanced pools of dNTPs. This leads to loss of DNA replication and repair, DNA damage, and apoptosis leading to a sustained IFN production (53). SAMHD1 is upregulated in response to viral infections. It plays a role in the antiviral immune response through initiation of the interferon pathway (51). *SAMHD1* disease-causing variants can present with AGS, SLE, and chilblain lupus (29, 30). SAMHD1 has been shown to be reduced or absent in the cells of patients with AGS (54). Cells from patients with *SAMHD1* mutations exhibited increased dNTP pools and DNA damage resulting in a failure of the cell cycle and cellular senescence. Increased DNA damage leads to upregulation in IFN-stimulated genes by activation of the innate immune system (53).

RNaseH2 (Ribonuclease H2) is a nucleic acid repair surveillance enzyme expressed in all cells and functions to remove ribonucleotides from DNA hybrid complexes. Three different genes encode the three protein components of the RNaseH2 complex, *RNASEH2A, RNASEH2B* and *RNASEH2C*. Mutations in all three genes have been associated with AGS and SLE. A recent study established mice models in which mutations in *RNASEH2B* trigger an increase of the expression levels of ISGs. The results of this work also proposed that this inflammatory response is dependent upon the cGAS/STING pathway (28). The findings of another study described that these mutations result in accumulation of ribonucleotides in genomic DNA placed during replication, which causes chronic DNA damage triggering the p53 pathway and type I IFN production (55). It has also been reported that fibroblasts from patients with SLE and AGS secondary to RNaseH2 mutations, as well as RNaseH2 deficient mice have significant accumulation of ribonucleotides and increased DNA in the cytoplasm. Furthermore, patient fibroblasts revealed an upregulation in IFN stimulated genes, which was enhanced, among other factors, by UV light exposure. UV light is a major trigger of SLE symptoms and it is thought that UV can raise apoptotic debris containing nucleic acids. Therefore, a deficiency in RNAseH2 in individuals exposed to UV light could be a possible link between genetic and environmental factors in the pathogenesis of SLE (55). Approximately one-third of AGS patients with variants in the RNaseH2 complex have positive ANA. Sequencing of the genes encoding the RNaseH2 complex in 600 SLE subjects identified 18 rare variants. Clinically, these patients mainly showed cutaneous involvement, photosensitivity, arthritis, lymphopenia, and autoantibody formation; internal organ involvement was less frequent (41).

*ADAR1* (adenosine deaminase, RNA specific) gene, located on chromosome 1, encodes a widely expressed enzyme which is involved in the editing of long double strand RNA. Mutations in this gene have been reported to cause AGS and SLE (7, 8). A proinflammatory signal upon recognition of viral or cellular dsRNA unedited due to loss of function mutations in *ADAR1* has been described (56). Interestingly, mutations in this gene also cause Dyscrhromatosus Symmetrica Hereditaria (DSH1), a rare skin condition which has been associated with SLE (57).

ISG15 is transcriptionally regulated by IFN-alpha and beta. Patients with ISG15 deficiency have increased risk of viral infection. Mutations in *ISG15* are associated with central nervous system (CNS) disease including intracranial calcifications and seizures (20). Patients show immunological and clinical signs of enhanced IFN-alpha/beta immunity. Interestingly, mutations in *ISG15* have been found in AGS and, in addition, a higher expression of this gene in SLE patients has been reported in several studies (58–60) ISG15 negatively regulates IFN-alpha and beta production (58), and is known as an activator of natural killer (NK) cells and a driver of IFNγ secretion (61). Therefore, ISG15 has emerged as a potentially critical bridge between type I and type II IFN immune responses.

### Spondyloenchondrodysplasia

Spondyloenchondrodysplasia (SPENCD) is a rare immuno-osseous disorder which causes skeletal dysplasia as well as variable neurologic manifestations (spacticity, developmental delay, intracranial calcification). In addition, it has been reported that SPENCD patients also may show overlapping features of lupus such as nephritis, thrombocytopenia, and dsDNA antibodies amongst others. Indeed, some of these patients fulfilled the American College of Rheumatology classification criteria for lupus (6, 62). The *ACP5* gene encodes tartrate-resistant acid phosphate (TRAP) protein which is mostly expressed in monocytic cells including osteoclasts, macrophages, and dendritic cells (63). Several different biallelic null mutations in *ACP5* distributed throughout the protein have been identified in individuals diagnosed with SPENCD-associated lupus. All mutations identified to date, cause a complete loss of enzymatic function. TRAP regulates the phosphorylation levels of osteopontin (OPN) which is a cytokine required for the production of type I IFN by plasmacytoid dendritic cells in response to TLR9 stimulation (64). It has been described that decreased expression of TRAP triggers increased phosphorylation of OPN leading to overproduction of type I interferon (6, 62). In addition, after TLR9 stimulation, a reduced expression of TRAP provokes higher levels of IFN-alpha, interleukin-6, ISGs, and tumor necrosis factor (TNF) (65). It is worth to note that in a recent study *ACP5* was sequenced in nearly 1,000 SLE patients and more than 500 healthy controls. The results of this study showed a significant increase in heterozygous *ACP5* missense variants in SLE patients compared to healthy individuals (65).

## Complement deficiencies

The classical complement pathway begins with C1, which consists of C1q, two C1r molecules, and two C1s molecules. C1 binds to the Fc portion of IgG or IgM antibody which complexes to antigens. The binding results in the activation of C1q which activates C1r, and then activates C1s. C1s cleaves C4 (to C4a and C4b) and C2 (to C2a and C2b). C4b and C2b combine and cleave C3 which is added to the complex resulting in C4bC2bC3b (also known as C5 convertase). This complex will cleave C5 resulting in the assembly of the membrane attack complex (C5bC6C7C8C9). The alternative complement pathway begins with the hydrolysis of C3 to C3(H2O). C3(H2O) is always present to a small degree but is maintained in homeostasis. The alternate pathway is initiated when C3(H2O) binds to factor Bb (factor B having been cleaved by factor D to form factor Ba and Bb) which forms C3b(H2O)Bb. The C3b(H2O)Bb is an alternative C3 convertase (66). Any defect in these complement components might prevent or hinder the removal or clearance of apoptotic cells or immune complexes, thus allowing these potential autoantigens to activate the immune system and lead to a loss of tolerance. Therefore, deficiencies of C1q, C1r, C1s, C2, or C4 have been strongly associated with both immunodeficiency as well as autoimmunity, including lupus-like phenotype (67).

Approximately 90% of people with C1q deficiency develop lupus like-phenotype (9, 68) including clinical characteristics such as photosensitive skin rash, nephritis, oral ulceration, and arthritis. Most of these patients have early onset disease with an age range from 6 months to 42 years, and a median age of onset of 6 years (69). Interestingly, patients with C1q deficiency have normal complement C3 and C4 levels with low total hemolytic complement levels which can be a helpful tool in diagnosis (70). Of interest, there are case reports of treatment with plasma transfusion, which restores C1q levels, leading to resolution of symptoms (71). Another small case series reported successful cure of patients with a C1q deficiency with bone marrow transplantation (72). C1q is encoded by three genes (*C1QA, C1QB*, and *C1QC*) which are closely linked on chromosome 1p34-1p36 (69). Multiple causal mutations, in the three genes, resulting in the deficiency of C1q have been identified in individuals with SLE-like phenotype. C1q is directly responsible for identification and opsonization of apoptotic cells which stimulates phagocytosis and activates the classical complement pathway. Apoptosis generates cellular debris, which if not properly cleared, can promote autoimmunity. Thus, deficiency of C1q results in autoantigen presentation with subsequent loss of tolerance (44). In addition, C1q suppresses IFN alpha production by interacting with leucocyte associated Ig-like receptor (LAIR)1 on plasmacytoid dendritic cells, and indirectly through uptake of C1q containing immune complexes by monocytes rather than plasmacytoid dendritic cells which are the primary producers of IFN alpha (68). Furthermore, C1q can inhibit TLR7 and TLR9 induced IFN-alpha production. Consequently, it has been described that patients with C1q deficiency develop increased levels of IFN alpha (9, 68). Taken into consideration the IFN-signature found in these patients, complement deficiencies could be also considered as secondary interferonopathies.

Deficiencies in C1r and C1s are rare and these patients usually die at a young age due to recurrent and severe infections. However, more than a half of these patients develop a lupus-like disease, with skin involvement and ANA positivity being the most noticeable features (73). Both genes encoding C1s and C1r are located on the short arm of chromosome 12 and several deleterious mutations, resulting in no detectable protein in the serum, have been identified in patients with lupus-like phenotype. Remarkably, consistent reduction in the serum protein levels of C1s in patients with C1r deficiency and low levels of C1r in patients with C1s deficiency have been observed (10, 11, 74).

C2 is the most common complement deficiency, occurring in about 1 in 20,000 individuals of European descent, however lupus develops only in about 10% of patients with C2 deficiency (73). This is likely due to the fact that C2 can be bypassed by the alternative complement pathway and is therefore not required for activation of the complement system (12). Patients with SLE secondary to C2 deficiency are similar in presentation and severity to the general SLE population, with a mean age of onset of 39 years. These patients will typically develop multiple infections at an early age, but are otherwise phenotypically similar to other patients with SLE (75).

C4 is a key component of the classical complement pathway. Homozygous deficiency of C4 results in a dysfunctional immune response which can cause lupus with >75% of penetrance (76). This protein is encoded by two genes, *C4A* and *C4B*, closely located within the human major histocompatibility complex (MHC) on chromosome 6. There is a complex pattern of variation in this region and duplications of C4 genes are common. The copy number variation (CNV) of these genes range from two to eight copies (13). Interestingly, the relationship of C4 gene copy number with non-Mendelian SLE has been repeatedly analyzed in different populations and the results of these studies consistently reported that the fewer the number of gene copies the higher the risk of lupus. Conversely, an increased number of C4 gene numbers is protective (77, 78). A deficiency in C4 in mice alters B cell tolerance by increasing the number of self-reactive B cells. These mice develop lupus-like features like glomerulonephritis and high levels of autoantibodies (79).

## Autoimmune lymphoproliferative syndrome

Autoimmune lymphoproliferative syndrome (ALPS) is a rare autoimmune disease mainly caused by mutations in FAS-mediated apoptotic pathway genes (17, 18). ALPS patients present with clinical features similar to SLE and mutations in these genes have been associated with both diseases (17, 18). The fas cell surface death receptor (FAS) is a protein in the TNF receptor superfamily. It plays a key role in programmed cell death; the binding of this receptor with its ligand results in signaling complex that includes Fas-associated death domain protein (FADD), caspase 8, and caspase 10 (17). Apoptosis is of particular interest in lupus as abnormalities in this process provide a source of autoantigens which are thought to drive the autoimmune response in this disease. Apoptotic cell death results in increased DNA fragments, which if not properly processed can accumulate and result in autoimmunity. *FAS* gene polymorphisms have been shown to be associated with SLE (80) and variants in the *FASL* gene have been related to increase apoptosis (81). In this context, it is noteworthy that mice with deficiencies in Fas and FasL develop clinical features similar to SLE and ALPS, thus represent useful murine models to study the pathophysiology of both diseases. Specifically, MRL/*lpr* mice have been widely used to investigate lupus and these studies significantly contributed to our current knowledge of the pathogenesis of SLE (82, 83).

## Other genes involved in monogenic lupus

PRKCD (protein kinase c delta) is a signaling kinase with multiple downstream target proteins which plays a role in regulating B cell development, proliferation, and apoptosis (84). The absence of PRKCD results in chronic B cell receptor signaling, decreased apoptosis, and increased response to stimulation. Conversely, overexpression of PRKCD results in inhibition of cell growth (85). A mutation in the *PRKCD* gene has been identified in a family with monogenic SLE, and is associated with loss of B cell tolerance and an increased number of immature B cells even in family members heterozygous for the mutation (24). In addition, PRKCD has a negative role in T cell proliferation and a deficiency in PRKCD results in increased T cell activation contributing to T cell autoimmunity (84). Patients with SLE secondary to *PRKCD* mutation demonstrate typical features of lupus including autoantibody production as well as increased incidence of glomerulonephritis.

Both *DNASE1* (deoxyribonuclease 1) and *DNASE1L3* (deoxyribonuclease 1 like 3) genes encode proteins involved in the nucleic acid degradation pathway. DNASE1L3 enzyme plays an important role in the clearance of DNA debris from apoptotic cells and exogenous DNA. A loss of function variant in this gene has been identified as the cause of a monogenic form of lupus. Positive ANA, anti-dsDNA, and hypocomplementemia among other features were present in all lupus patients harboring this variant (16). DNASE1L3 variations have also been reported in patients with hypocomplementemic urticarial vasculitis syndrome (86). A heterozygous null allele in *DNASE1*, which encodes for an endonuclease with certain degree of homology to DNASE1L3, has also been identified in individuals diagnosed with a monogenic form of SLE (15). Further studies have also reported the association of polymorphisms in *DNASE1* with non-Mendelian SLE (87). All these findings are consistent with the demonstration that mice deficient in DNASE1 develop a lupus-like phenotype (88). Interestingly, mutations in *DNASE2*, which encodes another member of the DNAse family, have been identified in three children presenting with severe autoimmune features. Although these patients did not fulfill criteria for a classification of SLE, all of them showed high levels of anti-DNA antibodies among others lupus-like symptoms (89).

Others monogenic disorders presenting rare cases of lupus-like phenotype have been reported. Although in most of them the mechanism causing autoimmunity is unclear, several causal genes have been described (Table 1), including *CYBB* gene causing chronic granulomatous disease, *PTPN11* and *SHOC2* genes associated with Noonan Syndrome, among others. Further studies focused on understanding the role of these genes in autoimmunity will help to better understand the pathogenesis of SLE.

## Final considerations

**Figure 2:**
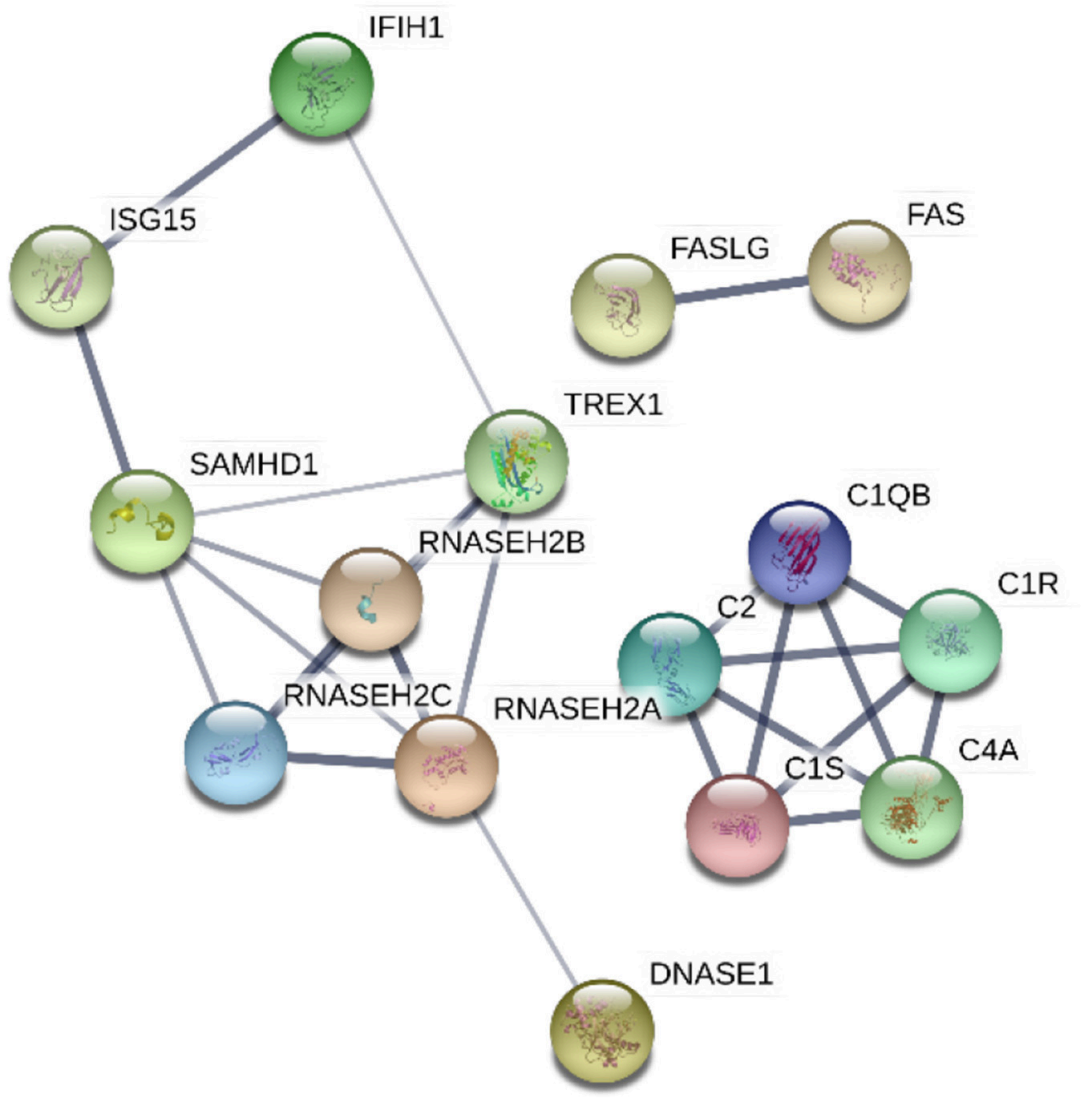
Protein-protein interaction network of proteins encoded by genes found to cause monogenic lupus. The confidence of data supporting these interactions are represented by the thickness of the lines. The analysis was performed using STRING V10.0 (STRING https://string-db.org/).

Understanding the implications of the genes identified to cause monogenic lupus have enhanced our knowledge of pathways and molecular mechanisms involved in the pathogenesis of SLE. As it has been explored through this review, monogenic lupus results from mutations in genes related to the immune response, either in the innate or in the adaptive immune system. Furthermore, protein-protein interaction analysis suggests that these genes encode proteins with related functions, creating molecular networks (Figure 2). Specifically, these genes are primarily involved in pathways including the complement system, nucleic acid repair, nucleic acid degradation and sensing, apoptosis, and type I interferon regulation. Although we do not yet know the full extent of monogenic lupus, the study of this type of lupus has provided new areas of investigation applicable to non-Mendelian SLE. Many genes have been identified as causes of monogenic lupus and at the same time have been associated with common forms of SLE, such as C4 number variation and polymorphisms in *TREX1*, among others. Besides, a recent study proposes that a set of rare variants across *PRKCD* play a role in a wider context of SLE susceptibility (90). Altogether, these findings reinforce the idea of analyzing the genetics of complex diseases by taking into consideration their Mendelian forms, and highlight the potential contribution of rare variants to the heritability of SLE.

The molecular complexity of autoimmune diseases and the clinical overlap among them makes accurate diagnosis and specific targeted therapy more challenging. In this context, a better knowledge of the genetic bases may generate insights into biomarker development and new drug targets.

## Author contributions

All authors listed have made a substantial, direct and intellectual contribution to the work, and approved it for publication.

### Conflict of interest statement

The authors declare that the research was conducted in the absence of any commercial or financial relationships that could be construed as a potential conflict of interest.
